# Activation of GABA type A receptor is involved in the anti-insomnia effect of Huanglian Wendan Decoction

**DOI:** 10.3389/fphar.2024.1389768

**Published:** 2024-05-23

**Authors:** Liang Li, Xiaorong Wu, Jili Gong, Zhuqiang Wang, Weibo Dai, Li Qiu, Hongyuan Zuo, Mengqin Yi, Hui Yuan, Mei Hu, Zhaobing Gao, Fuyun Tian

**Affiliations:** ^1^ Pharmacology Laboratory, Zhongshan Traditional Chinese Medicine Hospital, Guangzhou University of Chinese Medicine, Zhongshan, China; ^2^ School of Pharmaceutical Sciences, Guizhou Medical University, Guiyang, China; ^3^ Zhongshan Institute for Drug Discovery, Zhongshan, China; ^4^ School of Pharmacy, Zunyi Medical University, Zunyi, China

**Keywords:** Huanglian Wendan Decoction (HWD), GABA A type receptor (GABAAR), insomnia, zebrafish, traditional Chinese medicine systems pharmacology (TCMSP)

## Abstract

Huanglian Wendan Decoction (HWD) is a traditional Chinese medicine (TCM) prescribed to patients diagnosed with insomnia, which can achieve excellent therapeutic outcomes. As positively modulating the γ-aminobutyric acid (GABA) type A receptors (GABAARs) is the most effective strategy to manage insomnia, this study aimed to investigate whether the activation of GABAARs is involved in the anti-insomnia effect of HWD. We assessed the metabolites of HWD using LC/MS and the Traditional Chinese Medicine Systems Pharmacology (TCMSP) database and tested the pharmacological activity *in vitro* and *in vivo* using whole-cell patch clamp and insomnia zebrafish model. In HEK293 cells expressing α1β3γ2L GABAARs, HWD effectively increased the GABA-induced currents and could induce GABAAR-mediated currents independent of the application of GABA. In the LC-MS (QToF) assay, 31 metabolites were discovered in negative ion modes and 37 metabolites were found in positive ion modes, but neither three selected active metabolites, Danshensu, Coptisine, or Dihydromyricetin, showed potentiating effects on GABA currents. 62 active metabolites of the seven botanical drugs were collected based on the TCMSP database and 19 of them were selected for patch-clamp verification according to the virtual docking simulations and other parameters. At a concentration of 100 μM, GABA-induced currents were increased by (+)-Cuparene (278.80% ± 19.13%), Ethyl glucoside (225.40% ± 21.77%), and β-Caryophyllene (290.11% ± 17.71%). In addition, (+)-Cuparene, Ethyl glucoside, and β-Caryophyllene could also serve as positive allosteric modulators (PAMs) and shifted the GABA dose-response curve (DRC) leftward significantly. In the PCPA-induced zebrafish model, Ethyl glucoside showed anti-insomnia effects at concentrations of 100 μM. In this research, we demonstrated that the activation of GABAARs was involved in the anti-insomnia effect of HWD, and Ethyl glucoside might be a key metabolite in treating insomnia.

## 1 Introduction

Insomnia is a prevalent sleep disorder characterized by difficulty falling or staying asleep or enduring non-restorative sleep. With a prevalence of 30%, insomnia is now a significant health and economic burden worldwide (Buysse, 2013; Morin et al., 2015; Vargas et al., 2020). This disorder is usually associated with medical and mental disorders, including fatigue, reduced energy, impaired attention, and mood disturbances, and is one of the most common complaints in medical practice. In addition, insomnia is related to a variety of other disorders, including depressive disorder (Baglioni et al., 2011), anxiety disorder (Johnson et al., 2006), hypertension (Vgontzas et al., 2009), and suicidality (Pigeon et al., 2012), and recurrent insomnia increases the risk of mortality (Parthasarathy et al., 2015).

In clinical practice, insomnia is most often managed with medications, and positively modulating the γ-aminobutyric acid (GABA) type A receptors (GABAARs) is the most effective strategy (Buysse, 2013). GABA binding can activate the GABAARs, which mediate the influx of chloride, controlling the resting membrane potential. GABAARs are pentameric ligand-gated chloride channels, which are mostly heteropentamers assembled from numerous subunits, including α1–6, β1–3, γ1–3, δ, ε, π, θ and ρ1–3. The constitute of GABAARs is variable, and the function usually depends on the alpha subunit it contains (Ghit et al., 2021). The α1 subunit encoded by the GABRA1 gene is most ubiquitously expressed in most brain regions and is closely associated with circadian rhythm and sleep regulation (Jacob et al., 2008). In sleep-awake regulation, GABAARs activation promotes sleep by inhibiting the arousal systems (Saper et al., 2010; Buysse, 2013). GABAARs modulators used for treating insomnia can be divided into Benzodiazepines (BDZ) and non-BDZ, both enhance GABA-mediated inhibition through allosteric modulation of the GABAARs (Downing et al., 2005; Solomon et al., 2019). These agents are most effective for treating sleep problems and many of them are approved by the FDA for the treatment of insomnia, such as diazepam (Morin et al., 2015). However, these agents might be problematic for drug dependence and drug abuse (Krystal, 2009), and alternative treatments are needed.

**FIGURE 1:**
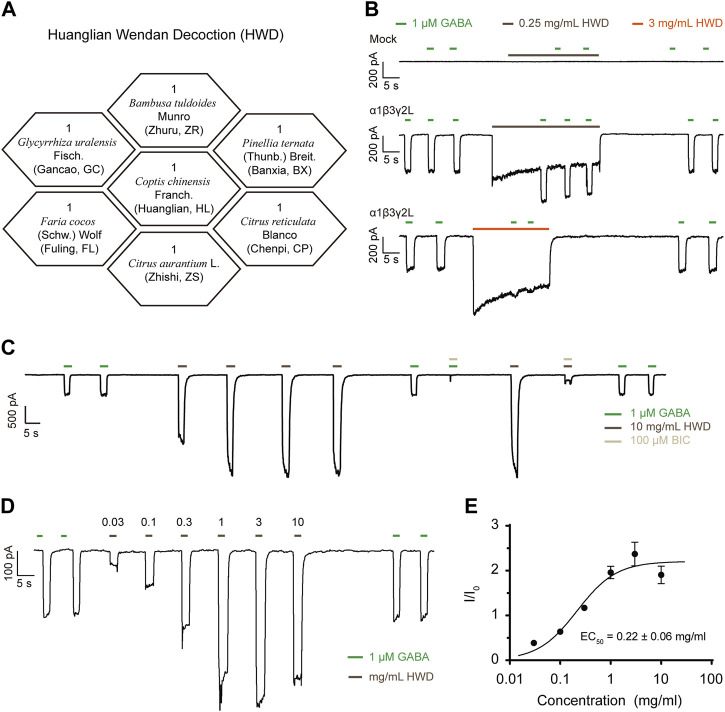
HWD served as a positive modulator of α1β3γ2L GABAARs. **(A)** The botanical drugs composition of HWD. **(B)** The effects of HWD on HEK293 cells expressing α1β3γ2L GABAARs. **(C)** The effects of BIC on HWD- or GABA-induced currents. **(D)** Representative trace induced by HWD of different concentrations. **(E)** DRC of the agonist effect of HWD.

Huanglian Wendan Decoction (HWD) is a traditional Chinese medicine (TCM) derived from “six-factor syndrome differentiation” and is mainly used for clearing heat and removing annoyance, removing dampness, and resolving phlegm. HWD formula consists of seven main botanical drugs, including Coptis chinensis Franch. [Ranunculaceae; Coptis Rhizoma] (Huanglian, HL), Pinellia ternata (Thunb.) Breit. [Araceae; Pinelliae Rhizoma] (Banxia, BX), Citrus aurantium L. [Rutaceae; Aurantii Fructus Immaturus] (Zhishi, ZS), Citrus reticulata Blanco [Rutaceae; Citri Reticulatae Pericarpium] (Chenpi, CP), Faria cocos (Schw.) Wolf [Polyporaceae; Poria] (Fuling, FL), Bambusa tuldoides Munro [Poaceae; Bambusae Caulis in Taenias] (Zhuru, ZR), and Glycyrrhiza uralensis Fisch. [Fabaceae; Glycyrrhizae Radix et Rhizoma] (Gancao, GC) (Figure 1A). Studies have confirmed that HWD could be mainly used to treat vascular and metabolic disorders, including atherosclerosis and type 2 diabetes, suggesting its regulation in glucose and lipid metabolism (Li et al., 2021; Shi et al., 2022). In our clinical practice, we prescribe HWD to patients diagnosed with insomnia, which can achieve excellent therapeutic outcomes (Li et al., 2022), suggesting that HWD can be used as an alternative treatment option for insomnia. However, the potential mechanism of HWD for insomnia is not well defined. Based on the close correlation between GABAARs and insomnia, we propose a hypothesis that the effects of HWD on GABAARs were involved in the possible mechanisms of the anti-insomnia effect of HWD. This study accumulates the theoretical basis for the further rational application of HWD and lays the foundation for future clinical research.

## 2 Methods

### 2.1 Freeze-dried powder

Traditional Chinese medicine (TCM) decoction pieces used in the experiment were purchased from the Chinese herbal pharmacy of Zhongshan Traditional Chinese Medicine Hospital and certified as genuine by Chief Chinese Pharmacist Weibo Dai. The seven TCM pieces of Huanglian Wendan Decoction are Coptis chinensis Franch. [Ranunculaceae; Coptis Rhizoma], Pinellia ternata (Thunb.) Breit. [Araceae; Pinelliae Rhizoma], Citrus aurantium L. [Rutaceae; Aurantii Fructus Immaturus], Citrus reticulata Blanco [Rutaceae; Citri Reticulatae Pericarpium], Faria cocos (Schw.) Wolf [Polyporaceae; Poria], Bambusa tuldoides Munro [Poaceae; Bambusae Caulis in Taenias], and Glycyrrhiza uralensis Fisch. [Fabaceae; Glycyrrhizae Radix et Rhizoma].

According to the decocting process of traditional Chinese medicine, 200 g of each of the above seven TCM decoction pieces was mixed, and 14 L of water was added to soak the TCM decoction pieces for 30 min. After that, the TCM decoction pieces were boiled over the fire and simmered with the gentle fire for 1 h. After pouring out the medicinal liquid of the 1st decoction, the dregs of the TCM decoction pieces were added to 14 L of water to decoct for the second time, and the time of the 2nd decoction was the same as that of the 1st decoction. After the 2nd decoction was combined and filtered through gauze, it was concentrated to 1 g/mL by rotary evaporator, quickly added liquid nitrogen, and placed in a vacuum freeze-drying machine (freeze-drying temperature −45°C, vacuum degree of 1,000 Pa) to dry for 72 h. The lyophilized powder of HWD solution obtained from the preparation was quickly transferred to a self-sealing bag, and placed in a low-temperature, dry, and light-avoiding place for storage.

### 2.2 Chemicals

Four- Chloro-DL-phenylalanine (PCPA) was purchased from the Harveybio (Beijing, China). Melatonin was purchased from Aladdin (Shanghai, China). Stiripentol was purchased from Macklin (Shanghai, China). All active metabolites of the botanical drugs were purchased from Topscience (Shanghai, China).

### 2.3 LC/MS

The lyophilized powder of HWD was dissolved in 20% methanol-water by ultrasonication, and the solution with a concentration of 78.3 mg/mL was filtered by 0.22 μm microporous membrane, then put into a liquid injection vial and stored at 4°C for testing. Metabolites were identified based on accurate molecular weight combined with secondary spectrum fragment analysis and an Agilent TCM database. Chromatographic conditions: LC 1290-UPLC (Agilent, USA) with a Zorbax eclipse Plus C18 column (150 mm × 3.0 mm, 1.8 µm) and mobile phase consisting of ultrapure water containing 0.1% formic acid (A) and acetonitrile(B); sample volume: 0.5 μL; flow rate: 0.5 mL/min; column temperature: 30 °C; elution conditions: gradient elution program (0–2 min, 5% B; 2–20 min, 5%–35% B; 20–24 min, 35%–40% B; 24–25 min, 40%–50% B; 25–30 min, 50%–95%B; 30–35 min, 95% B; 35–38 min, 5% B). Mass spectrometry conditions: Q-TOF 6545 (Agilent, USA) high-resolution mass spectrometry system with ESI electrospray ion source; instrument settings: both negative and positive ion mode for detection; drying gas temperature: 300°C; drying gas flow rate: 8 L/min; Sheath gas temperature: 350 °C; Sheath gas flow rate: 11 L/min; atomizing gas pressure: 45 psi; cracking voltage: 130 V; capillary voltage: 4000 V (+)/3500 V (−); nozzle voltage: 0 V (+)/1000 V (−); primary mass scanning range: 100–1700 m/z; the scanning range of child ion: 20–1,200 m/z; the collision energy (CE): 10/20/40 V.

### 2.4 Zebrafish model

Wild-type AB strain zebrafish were purchased from China Zebrafish Resource Center, CZRC (Wuhan, China). Adult zebrafish were reared at 28°C on a 14/10 h light/dark cycle. Following natural spawning, eggs were collected and maintained in an embryo buffer under continuous light conditions at 28°C. The embryo buffer contained (in mM): 5 NaCl, 0.17 KCl, 0.33 MgSO_4_, and 0.33 CaCl_2_. At 5 days post fertilization (dpf), zebrafish larvae were pre-treated with fish water containing active metabolites, melatonin, or 1% DMSO for 2 hours and then were exposed to 10 mM PCPA for 8 h; the moving of the zebrafish larvae was recorded in the observation box and analyzed with Noldus EthoVision XT (Noldus XT, Wageningen, Netherlands).

### 2.5 Plasmid

Human GABRA1 and GABRB3 were cloned into pEG BacMam plasmids, respectively; a plasmid expressing human GABRG2 from the CMV promoter and EGFP from the EF1a promoter was constructed to ensure the presence of γ2 subunits based on the green fluorescence.

### 2.6 Cell culture and transfection

The Human embryonic kidney 293T (HEK293T) cell lines were grown in DMEM basal medium (Gibco) supplemented with 10% fetal bovine serum (FBS, Gibco) and maintained kept at 37°C in a 5% CO_2_ incubator. The cells were passaged the day before transfection at a ratio of 1:4. The transfections were performed using Lipofectamine 2000 (Thermo Fisher Scientific) according to the manufacturer’s instructions, and the fluid was changed 6 h after transfection. To express GABAARs, pcDNA3.1 plasmids containing wild-type GABRA1 were co-transfected with GABRB3 and GABRG2 at a ratio of 1:1:1.

### 2.7 Whole-cell patch-clamp recordings

Whole-cell patch-clamp recordings were performed at room temperature (22°C–25°C) using an Axon 700B amplifier and a Digidata 1550B digitizer (Axon Instruments, Molecular Devices). Pipettes were pulled with resistances of 3–7 MΩ when filled with the intracellular solution. The pipette solution contained (in mM): 153 KCl, 1 MgCl_2_, 10 HEPES, and 5 EGTA (280–290 mOsm), with pH adjusted to 7.4 using KOH. The extracellular solution contained (in mM): 142 NaCl, 8 KCl, 1 CaCl_2_, 6 MgCl_2_, and 10 HEPES (290–300 mOsm), with pH adjusted to 7.4 using NaOH. Metabolite or botanical drug powder solutions were applied through an RSC-200 Rapid Solution Changer (BioLogic, France). Recordings were performed at a holding potential of −50 mV. Voltage clamp mode was used and the signal was sampled at 10 kHz and low pass filtered at 2 kHz.

### 2.8 Screening active metabolites targeting GABRA1 in TCMSP

The information on the active metabolites that interact with GABRA1 was downloaded from the TCMSP database, and medicine, metabolites, and target information were visualized using Cytoscape. This network showed that the drugs, metabolites, and GABRA1 were expressed as nodes, whereas their interactions were as edges.

### 2.9 Molecular docking of active metabolites with GABRA1

PubChem was used to obtain 3D structures of metabolites. Chem 3D was used to optimize small molecules and export them to the MOL2 format. The RCSB Protein Data Bank (PDB) was used to obtain the 3D structure of GABRA1 in PDB format (7QNE), and PyMOL was used to analyze protein dehydration and hydrogenation. AutoDock software was used to convert the metabolite and target protein format to the PDBQT format. Finally, AutoDock Vina was run for virtual docking. It is generally accepted that the lower the energy is, the higher the binding possibility.

### 2.10 Statistical analysis

Patch-clamp data were analyzed using Clampfit 10.6 (Molecular Device, United States) and GraphPad Prism 8 (GraphPad Software, United States). Concentration-response curves were fitted using the following four-parameter Hill equation: Y=Bottom + (Top-Bottom)/(1 + 10^((LogEC_50_-X)*HillSlope)), where Bottom and Top represent the channel’s maximum and minimum responses to the metabolites, respectively; X is the value of the logarithm of the concentration, Y is the I_drug_/I_control_ value, EC_50_ is the concentration producing a half-maximum response, and Hillslope is the factor to describe the steepness of the curve. All results were presented as mean ± standard error of the mean (SEM). Statistical analyses were performed using Student’s t-test, *p < 0.05, **p < 0.01 and ***p < 0.001.

## 3 Results

### 3.1 HWD activated α1β3γ2L GABAARs expressed in HEK293 cells

The GABAAR α1 subunit encoded by GABRA1 is mainly associated with sleep regulation; thus, we co-transfected the GABRA1 gene in HEK293 cells with the GABRB3 and GABRG2 gene that is widely expressed in the brain and tested the effect of HWD freeze-dried powder on the α1β3γ2L GABAARs *in vitro*. We used GABA of 1 μM as ligands to induce current and pre-treated transfected cells with HWD for 15 s before co-treatment with GABA to ensure an adequate reaction between the active metabolites and the membrane receptors.

Interestingly, the application of HWD (freeze-dried powder) of 0.25 mg/mL or 3 mg/mL enhanced the GABA-induced currents significantly and induced an inward current itself (Figure 1B). Neither HWD- nor GABA-induced inward current was present in the mock control, suggesting that the current was mediated by α1β3γ2L GABAARs and HWD could serve as an agonist as GABA. In addition, HWD induced inward currents in cells expressed α2β3γ2L, α3β3γ2L, or α6β3γ2L (Supplementary Figure S1). We further analyzed the direct activation of HWD and found that HWD of 10 mg/mL could effectively induce currents independent of GABA (Figure 1C). The HWD-induced current was sensitive to bicuculline (BIC), an antagonist of GABAARs, which further demonstrated that the current induced by HWD was mediated by GABAARs rather than off-target effects on the background current of HEK293 (Figure 1C). In addition, the HWD concentration-response curve of agonist effect revealed a bell shape, the EC_50_ was 0.22 ± 0.06 mg/mL, with a maximum response at 3 mg/mL (Figures 1D,E), suggesting that the active metabolites in the HWD mixture may include both agonists and inhibitors.

### 3.2 Search for metabolites that affect GABRA1 in HWD by LC-MS and patch-clamp recording

To filter out the active metabolites in HWD, we tested the agonist effect of the seven botanical drugs, respectively. Surprisingly, all seven botanical drugs (freeze-dried powder) could induce a visible inward current in the HEK293 cells expressing α1β3γ2L GABAARs at a concentration of 10 mg/mL, and GC and ZR showed the most potent effects. (Supplementary Figure S1). Considering the general effectiveness of the seven botanical drugs, we performed an LC-MS (QToF) assay in positive and negative ion modes to identify the main metabolites in HWD rather than in a particular botanical drug. As shown in Supplementary Table S1, 2 31 metabolites were discovered in negative ion modes and 37 metabolites were found in positive ion modes.

**FIGURE 2:**
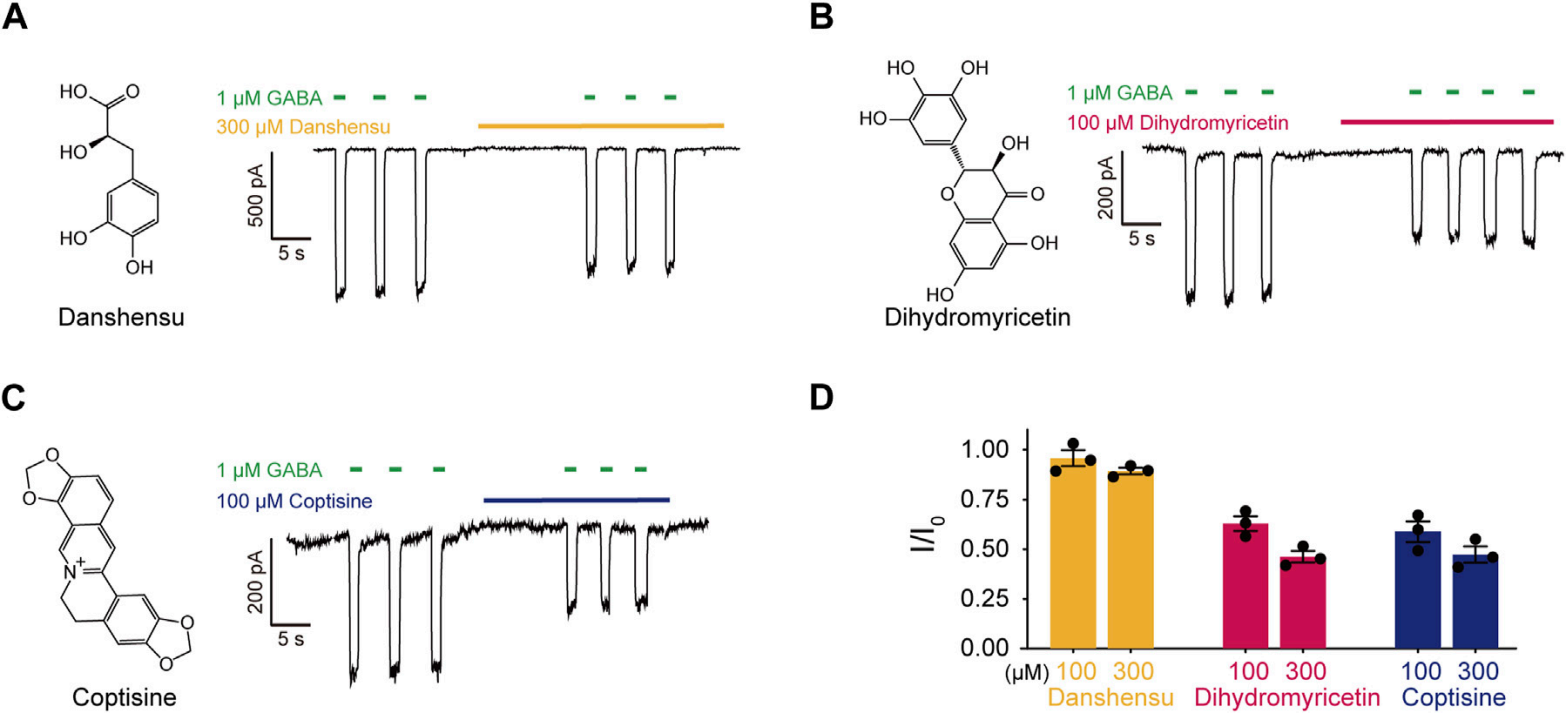
The effects of main metabolites in HWD on α1β3γ2L GABAARs. **(A–C)** Molecular structures and effects on α1β3γ2L GABAARs of **(A)** Danshensu, **(B)** Dihydromyricetin, and **(C)** Coptisine. **(D)** Statistic graph of the effects of Coptisine, Danshensu, and Dihydromyricetin on GABA-induced current.

We selected Coptisine, Danshensu, and Dihydromyricetin as representative metabolites to validate the effect of the main metabolites on α1β3γ2L GABAARs using patch-clamp electrophysiology. None of the three metabolites showed an agonist effect that induce a chloride current itself at concentrations of 100 μM or 300 μM. Interestingly, all of them displayed inhibitory effects. At a concentration of 100 μM, Coptisine and Dihydromyricetin showed significant inhibitory effects (I/I_0_ = 0.59 ± 0.05 and I/I_0_ = 0.63 ± 0.04, respectively); at a higher concentration of 300 μM, Danshensu slightly decrease GABA-induced current (I/I_0_ = 0.89 ± 0.02) (Figure 2), which might contribute to bell-shape concentration-response curve of HWD.

### 3.3 Search for metabolites that affect GABRA1 in HWD by network pharmacology

**FIGURE 3:**
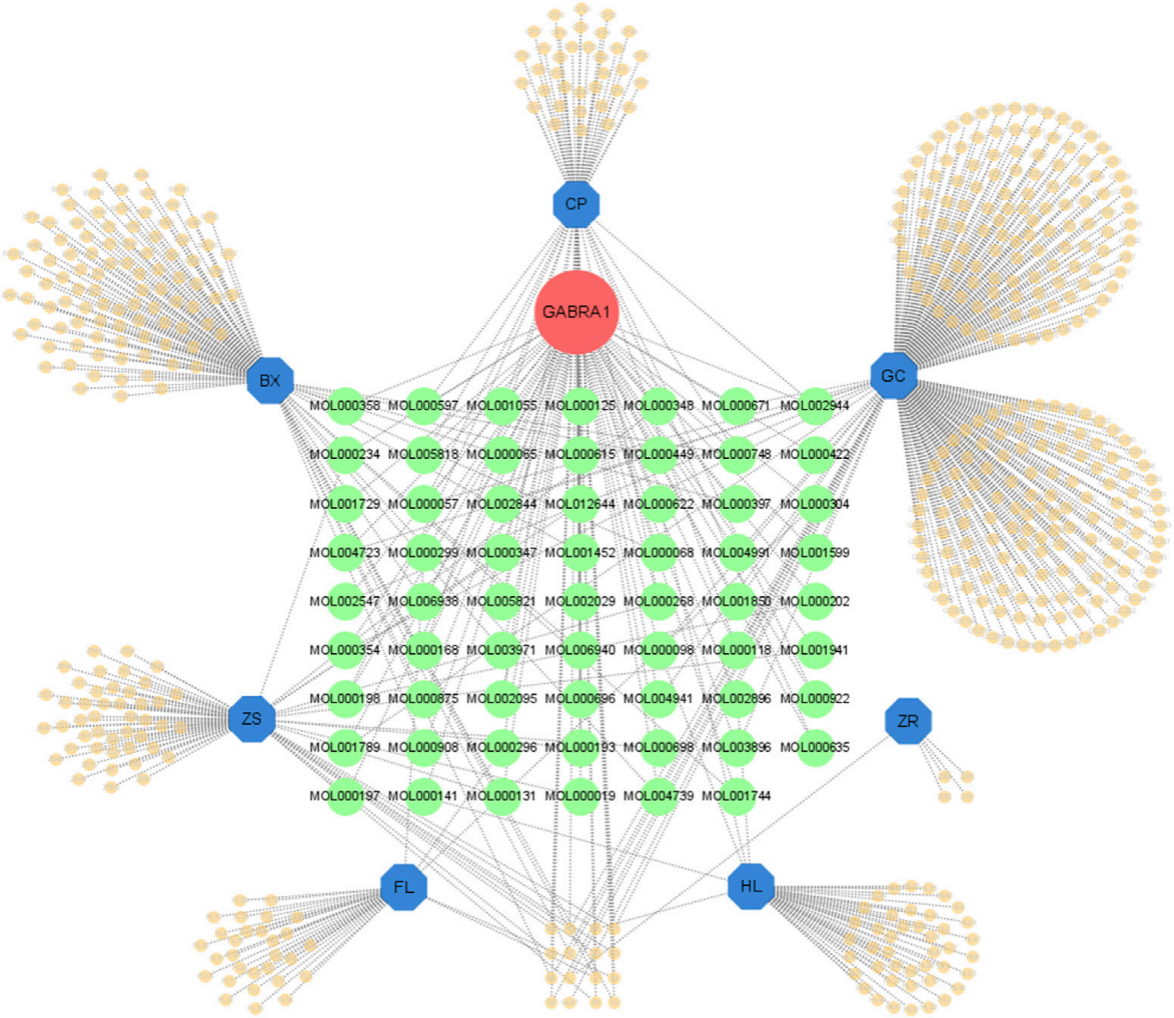
The botanical drugs-metabolites-GABRA1 network. Green circles represent metabolites that may act on GABRA1.

Network pharmacology has emerged as a valuable tool for analyzing complex systems in pharmacology studies; here, we tried to screen the active metabolites targeting GABRA1 in HWD using the TCMSP (Traditional Chinese Medicine Systems Pharmacology) database. Due to the general agonist effect of the seven botanical drugs (Supplementary Figure S1), we used all seven botanical drugs as keywords in the TCMSP database to obtain the active metabolites list and filtered out 62 active metabolites interacting with GABRA1 (Figure 3). As shown in Table 1, the 62 metabolites were sorted according to the binding affinity. The active metabolites list included 16 in BX, four in HL, 18 in CP, 15 in ZS, and 14 in RG, and several active metabolites were found in multiple botanical drugs, including quercetin (HL and GC), (−)-α-Terpineol (CP and GC), EIC (BX and ZS), and so on. However, no metabolites co-existed in all the seven botanical drugs, suggesting the existence of several potential modulators of GABAARs in HWD.

**TABLE 1:** Active metabolites of HWD targeting GABRA1.

MOL ID	metabolites	Binding affinity (kcal/mol)	OB (%)	BBB	DL	Botanical drugs
MOL001729	Crysophanol	−11.1	18.64	−0.2	0.21	Pinellia ternata
MOL000098	Quercetin	−10.3	46.43	−0.77	0.28	Coptidis Rhizoma、Radix Glycyrrhizae
MOL000615	Delta-amorphene	−10	17.95	1.99	0.08	Citri Reticulatae Pericarpium
MOL000449	Stigmasterol	−9.6	43.83	1	0.76	Pinellia ternata
MOL004991	7-Acetoxy-2-methyl isoflavone	−9.1	38.92	0.16	0.26	Radix Glycyrrhizae
MOL000296	Hederagenin	−9	36.91	0.96	0.75	Fructus Aurantii Immaturus
MOL003896	7-Methoxy-2-methyl isoflavone	−8.9	42.56	0.56	0.2	Radix Glycyrrhizae
MOL001941	Ammidin	−8.8	34.55	0.92	0.22	Fructus Aurantii Immaturus
MOL002844	Pinocembrin	−8.8	64.72	0.12	0.18	Radix Glycyrrhizae
MOL000358	β-sitosterol	−8.7	36.91	0.99	0.75	Pinellia ternata
MOL000354	Isorhamnetin	−8.2	49.6	−0.54	0.31	Radix Glycyrrhizae
MOL004941	(2R)-7-hydroxy-2-(4-hydroxyphenyl)chroman-4-one	−8.2	71.12	−0.25	0.18	Radix Glycyrrhizae
MOL000622	Magnograndiolide	−8.1	63.71	−0.24	0.19	Coptidis Rhizoma
MOL002896	Corydaldine	−8	49.3	0.56	0.09	Coptidis Rhizoma
MOL002029	(+)-Cuparene	−8	38.26	2.15	0.07	Citri Reticulatae Pericarpium
MOL001599	α-cubebol	−8	64.81	1.43	0.09	Radix Glycyrrhizae
MOL000422	Kaempferol	−8	41.88	−0.55	0.24	Radix Glycyrrhizae
MOL000597	Neryl acetate	−7.7	57.47	1.4	0.04	Citri Reticulatae Pericarpium
MOL000193	β-caryophyllene	−7.5	30.29	2.15	0.09	Fructus Aurantii Immaturus
MOL000908	β-elemene	−7.4	25.63	2.07	0.06	Pinellia ternata
MOL001789	Isoliquiritigenin	−7.4	85.32	−0.41	0.15	Radix Glycyrrhizae
MOL012644	Caryophellene	−7.3	23.79	2.08	0.09	Fructus Aurantii Immaturus
MOL000875	Cedrol	−7.2	16.23	1.46	0.12	Pinellia ternata
MOL000141	Hydroxytyrosol	−7.1	57.57	−0.17	0.03	Coptidis Rhizoma
MOL000635	Vanillin	−7	52	0.41	0.03	Citri Reticulatae Pericarpium
MOL000397	Cis-p-Coumarate	−6.9	45.98	0.36	0.04	Pinellia ternata
MOL000922	(R)-p-Menth-1-en-4-ol	−6.9	32.16	1.52	0.03	Citri Reticulatae Pericarpium
MOL001452	Protocatechualdehyde	−6.8	38.35	0.21	0.03	Pinellia ternata
MOL006938	DUR	−6.8	29.86	1.32	0.02	Pinellia ternata
MOL001055	5-isopropyl-2-methylbicyclo [3.1.0]hex-2-ene	−6.8	47.19	2.18	0.04	Citri Reticulatae Pericarpium
MOL002095	DEP	−6.8	52.19	0.57	0.07	Citri Reticulatae Pericarpium
MOL000019	D-Camphene	−6.7	34.98	2.19	0.04	Fructus Aurantii Immaturus
MOL000118	(−)-α-Terpineol	−6.7	48.8	1.72	0.03	Citri Reticulatae Pericarpium、Radix Glycyrrhizae
MOL001850	Isophorone	−6.6	44.98	1.66	0.03	Radix Glycyrrhizae
MOL000671	()-Menthol	−6.6	59.33	1.42	0.03	Radix Glycyrrhizae
MOL000202	Moslene	−6.5	33.02	2.05	0.02	Fructus Aurantii Immaturus
MOL000347	Syrigin	−6.5	14.64	−1.81	0.32	Fructus Aurantii Immaturus
MOL000234	L-Limonen	−6.4	38.09	2.13	0.02	Fructus Aurantii Immaturus
MOL000057	DIBP	−6.4	49.63	0.68	0.13	Citri Reticulatae Pericarpium
MOL002547	21,987_FLUKA	−6.4	40.92	2.14	0.04	Radix Glycyrrhizae
MOL004723	β-Terpinene	−6.4	42.29	2.12	0.02	Radix Glycyrrhizae
MOL000348	4-[(Z)-3-hydroxyprop-1-enyl]-2,6-dimethoxyphenol	−6.3	49.15	0.03	0.06	Fructus Aurantii Immaturus
MOL000698	(R)-(−)-alpha-Phellandrene	−6.3	27.51	2.17	0.02	Fructus Aurantii Immaturus
MOL000168	()-2-Carene	−6.2	46.69	2.27	0.04	Citri Reticulatae Pericarpium
MOL000696	β-terpineol	−6.2	47.89	1.48	0.03	Citri Reticulatae Pericarpium
MOL000125	(−)-α-Pinene	−6.1	46.25	2.3	0.05	Citri Reticulatae Pericarpium
MOL002944	(E)-Linalol pyranoxide	−6.1	44.25	0.94	0.04	Citri Reticulatae Pericarpium
MOL000131	EIC	−6	41.9	0.9	0.14	Pinellia ternata、Fructus Aurantii Immaturus
MOL000268	(1S,5S)-1-isopropyl-4-methylenebicyclo [3.1.0]hexane	−6	46.21	2.18	0.04	Fructus Aurantii Immaturus、Citri Reticulatae Pericarpium
MOL000299	Trimethyl citrate	−5.9	67.61	−0.33	0.07	Fructus Aurantii Immaturus
MOL001744	Uracil	−5.8	42.53	−0.08	0.02	Pinellia ternata
MOL000748	HMF	−5.6	45.07	−0.27	0.02	Pinellia ternata、Citri Reticulatae Pericarpium
MOL005818	2,5,5-trimethylhepta-1,6-diene	−5.6	44.34	2.07	0.02	Citri Reticulatae Pericarpium
MOL000304	Ethyl glucoside	−5.5	15.21	−3.15	0.06	Fructus Aurantii Immaturus
MOL000198	(R)-linalool	−5.5	39.8	1.36	0.02	Citri Reticulatae Pericarpium
MOL000197	Myrcene	−5.4	24.96	1.98	0.02	Fructus Aurantii Immaturus
MOL000068	L-Ile	−5.1	59.05	−0.11	0.02	Pinellia ternata
MOL003971	Threonin	−5	73.52	−2.56	0.01	Pinellia ternata
MOL000065	ASI	−5	79.74	−1.53	0.02	Pinellia ternata
MOL006940	D-2-Aminobutyrate	−4.8	68.78	−0.44	0.01	Pinellia ternata
MOL005821	(2S)-2-ethoxypentane	−4.6	39.6	1.94	0.01	Citri Reticulatae Pericarpium
MOL004739	DAL	−4.4	85.17	−0.51	0.01	Pinellia ternata

### 3.4 Verify the effect of the metabolites on GABRA1 using a patch-clamp method

**FIGURE 4:**
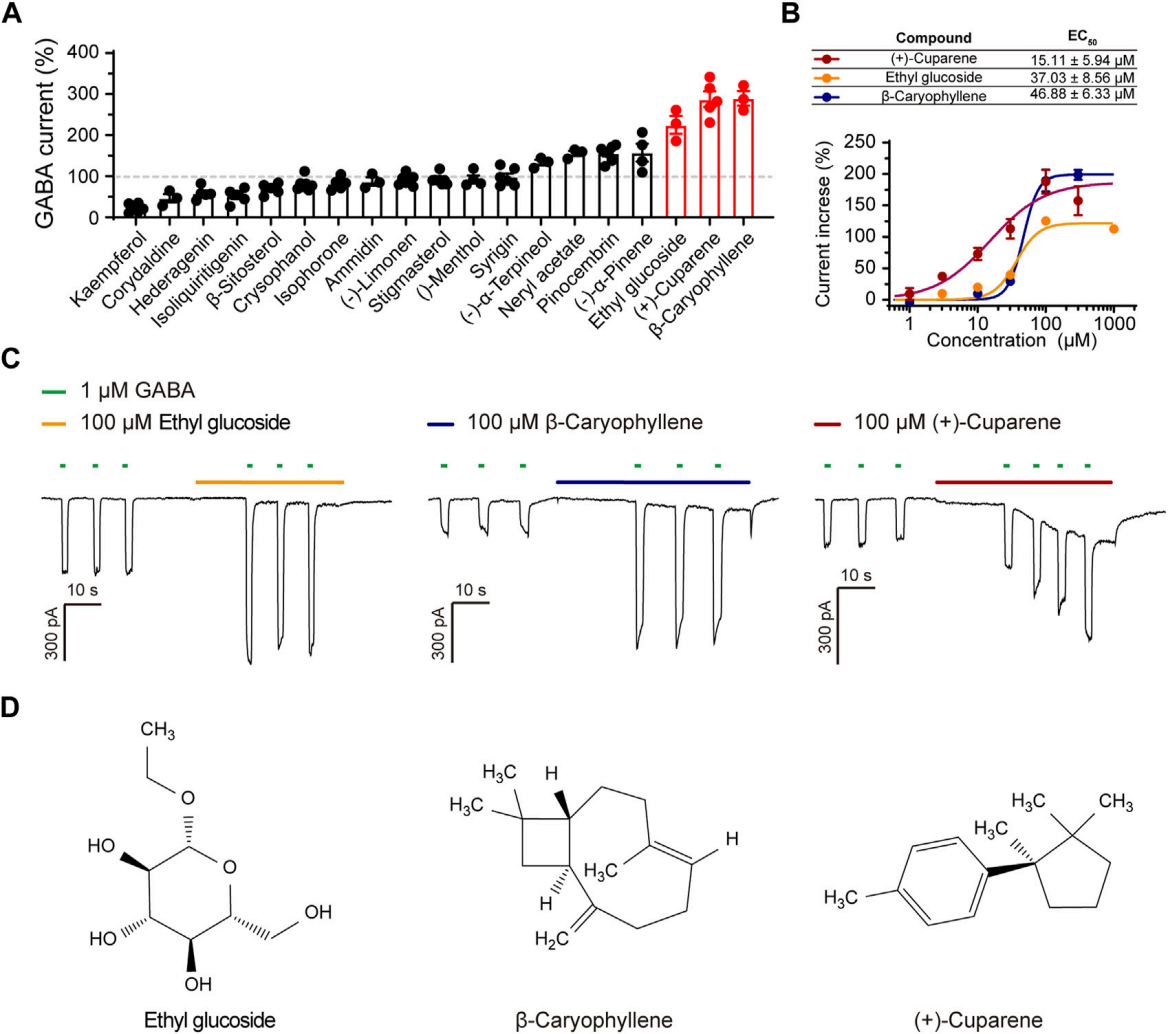
The effects of 19 active metabolites on α1β3γ2L GABAARs. **(A)** Statistic graph of the effects of 19 active metabolites on α1β3γ2L GABAARs; the test concentration was 100 μM. **(B)** DRC, **(C)** representative current trace, and **(D)** molecular structure of (+)-Cuparene, Ethyl glucoside, and β-Caryophyllene.

We applied oral bioavailability (OB), drug-likeness (DL), blood-brain barrier penetration, structural characterization, and easy availability as screening criteria to select active metabolites for the measurement of their activation on α1β3γ2L GABAARs using patch clamp (Table 1). Similar to the HWD test protocol, we used GABA of 1 μM as ligand and pre-treated transfected cells with metabolites for 15 s before co-treatment with GABA. After filtering, 19 molecules were chosen for patch-clamp evaluation. Among these metabolites, kaempferol and corydaldine suppressed the GABA-induced current by more than half at a concentration of 100 μM, which may explain the bell-shape DRC of the agonist effect of HWD (Figure 4A). Seven molecules effectively enhanced the GABA-induced current and three of them could increase the GABA current more than two-fold at a concentration of 100 μM, including β-Caryophyllene, (+)-Cuparene, and Ethyl glucoside (Figure 4). In addition, all the three molecules increased the GABA-induced current in a dose-dependent manner, and the EC_50_ was 15.11 ± 5.94 μM ((+)-Cuparene), 37.03 ± 8.56 μM (Ethyl glucoside), and 46.88 ± 6.33 μM (β-Caryophyllene), respectively (Figure 4B). Interestingly, (+)-Cuparene itself could also induce an inward current independent on the application of GABA, which may be responsible for the agonist effects of HWD (Figure 4C).

**FIGURE 5:**
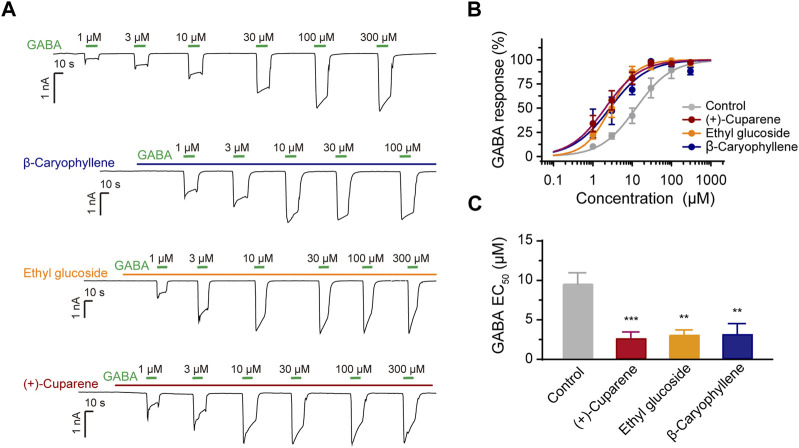
The PAM effects of three active metabolites on α1β3γ2L GABAARs. **(A)** Representative traces of GABA-induced currents after the application of the three active metabolites. **(B)** GABA DRC and **(C)** EC50 after the application of the three active metabolites.

We then tested whether the three metabolites could serve as PAMs of α1β3γ2L GABAARs. We pre-treated the HEK293 cells expressing α1β3γ2L with the test metabolites at a concentration of around EC_50_, and then co-treated with GABA of different concentrations to obtain a GABA dose-response curve (DRC). All three molecules shifted the GABA DRC leftward; the GABA EC_50_ was 9.35 ± 1.38 μM (control), 2.47 ± 0.88 μM ((+)-Cuparene), 2.99 ± 0.61 μM (Ethyl glucoside), and 3.06 ± 1.11 μM (β-Caryophyllene), respectively, demonstrating a PAM effect (Figure 5). In addition, β-Caryophyllene and (+)-Cuparene could increase the GABA-induced current in cells expressed α2β3γ2L, α3β3γ2L, or α6β3γ2L at a concentration of 30 μM; Ethyl glucoside showed weak effects on α2β3γ2L or α6β3γ2L and no effect on α3β3γ2L (Supplementary Figure S3).

### 3.5 Verify the anti-insomnia effect of metabolites in HWD using a zebrafish model

**FIGURE 6:**
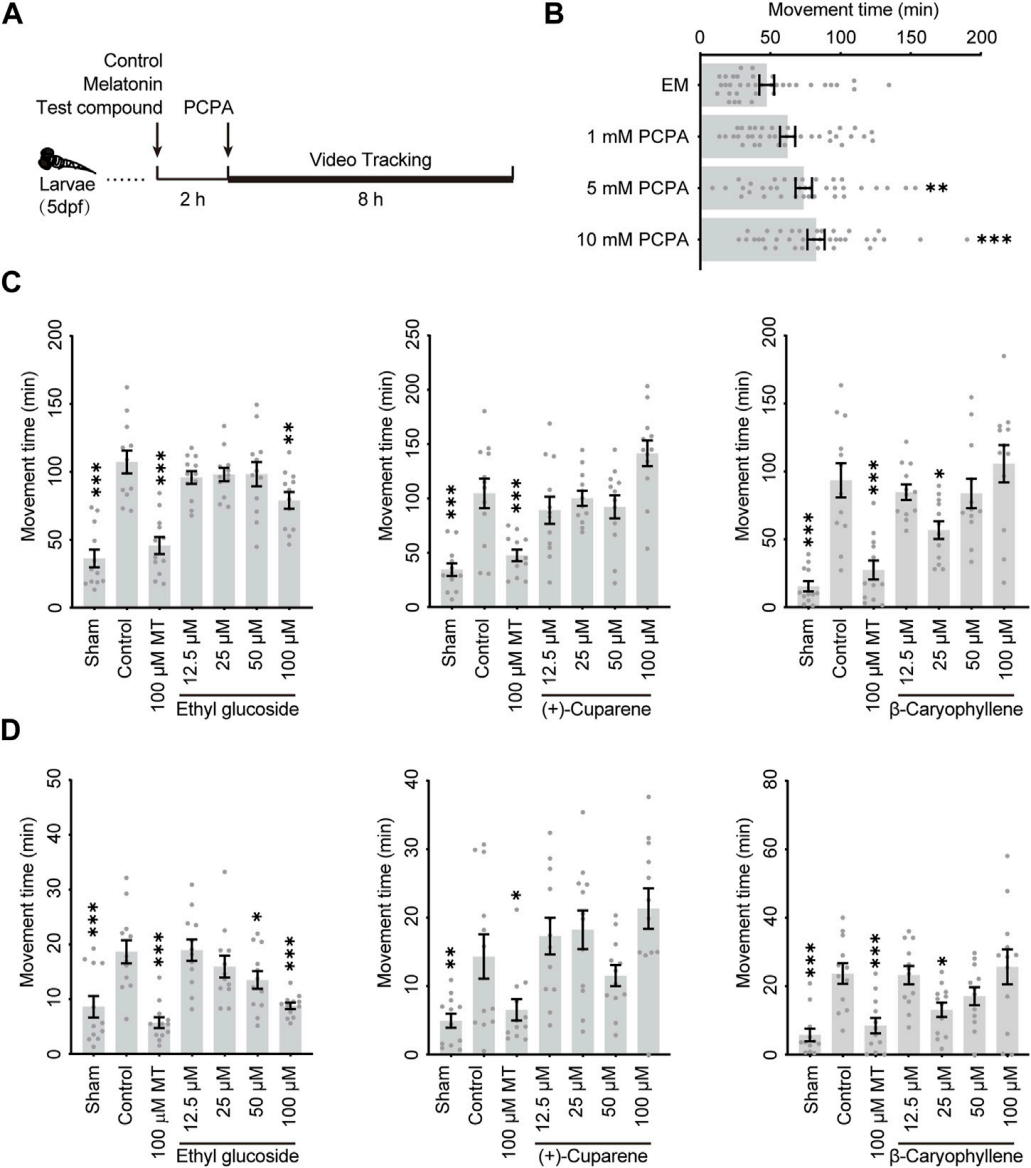
The anti-insomnia effect of three active metabolites in zebrafish model **(A)** Schema of the experimental regimen. **(B)** The dose-response relationship of PCPA The anti-insomnia effect of three active metabolites in the zebrafish model during **(C)** an 8-h recording or **(D)** the last 2 hours.

Zebrafish have been widely used to construct neurological disorder models for high-throughput drug screenings *in vivo*, which reduces the costs substantially compared to mammalian models. To verify the involvement of GABAARs in the anti-insomnia effects of HWD, we used para-chlorophenylacetic acid (PCPA)-induced insomnia zebrafish models to evaluate the hypnotic effects of the three most potent molecules. PCPA is a serotonin (5-HT) synthesis inhibitor that induces insomnia by disturbing the serotonergic system, which is necessary for sleep onset and maintenance. As shown in Figure 6B, the application of PCPA could increase the moving duration in a concentration-dependent manner, suggesting an insomnia-like behavior. Based on the dose-response relationship and the limited solubility, PCPA of 10 mM was used to establish the model, and melatonin was used as a positive control.

To evaluate the anti-insomnia effect of three metabolites, larvae of five dpf were treated with the test metabolite, control, or melatonin for 2 h. The larvae were exposed to PCPA to induce an insomnia-like phenotype, and their movements would be recorded by a camera and analyzed with tracking software (Figure 6A). As shown in Figure 6C, PCPA increased the movement duration significantly during an 8-h recording, and melatonin could effectively decrease insomnia. During an 8-h recording, Ethyl glucoside could relieve the hyperactive movement at a concentration of 100 μM, while β-Caryophyllene could relieve the hyperactive movement at a concentration of 25 μM. In addition, the decrements of movement duration were more significant during the last 2 hours (Figure 6D).

## 4 Discussion

HWD is a TCM that could be used in insomnia treatment. This study reveals that HWD can serve as a ligand of α1β3γ2L GABAARs, which may be responsible for their anti-insomnia effects. Based on the prediction of TCMSP and the confirmation using patch clamp, we find several monomeric metabolites as PAMs of α1β3γ2L GABAARs, which may be involved in the active metabolites of HWD in treating insomnia.

HWD can be an effective TCM recipe for treating insomnia, but the underlying mechanisms responsible for the anti-insomnia effects remain poorly defined. In a previous work, the mechanism of HWD against insomnia was investigated using a network pharmacology approach, demonstrating that this effect involves multiple signaling pathways, such as neurotransmitter signaling and NF-κB pathway (Dong et al., 2021). In our previous work on an insomnia rat model, several serum biochemical parameters were significantly changed, such as BDNF and GFAP; the concentration of several neurotransmitters in the brain was also different after the application of HWD, including GABA, 5-HT, and glutamate (Li et al., 2022). Here in this work, we report that HWD could activate GABAARs directly for the first time, which is consistent with the underlying mechanism of BZD, the most effective treatment against insomnia in clinical.

GABAARs are ligand-gated chloride channels that mediate fast neuronal inhibition in the brain. Based on their physiological roles, they are important drug targets for treating epilepsy, anxiety, and insomnia, and most of these drugs act through positive modulation of GABAARs. However, drugs targeting GABAARs often lead to side effects, including fatigue, dizziness, memory loss, or even mental symptoms such as anxiety, and some of these drugs can lead to tolerance and dependence. The effects of HWD in treating insomnia have been proven, demonstrating it can be used as replacement therapy in treating insomnia.

None of the three most potent metabolites, β-Caryophyllene, (+)-Cuparene, or Ethyl glucoside, have been reported as GABAARs positive modulators. The effects of β-caryophyllene on GABAARs have been tested before, but no increased effect of GABA-induced current was detected, possibly due to the different subunit composition (Janzen et al., 2021). β-caryophyllene has been reported as a neuropharmacological protector in seizure, anxiety, and depression, which may involve its effect on GABAARs (da Silva Oliveira et al., 2021). In addition, (−)-α-pinene was found to bind to aromatic residues of α1 and γ2 subunits of GABAA-BZD receptors in the molecular model (Yang et al., 2016), which was functionally verified in this work. Menthol and Stigmasterol have been reported as positive modulators of GABAARs (Watt et al., 2008; Karim et al., 2021), but they only showed slight effects at a concentration of 100 μM, which may be due to the different test systems and concentrations; both Stigmasterol and Menthol showed positive modulation at a concentration of 300 μM, and Stigmasterol also showed an agonist effect at this concentration (Supplementary Figure S2).

Zebrafish were widely used to establish neural disease models such as insomnia. Here, we first used PCPA to establish a novel insomnia model in zebrafish. PCPA can inhibit the synthesis of 5-HT by antagonizing tryptophan hydrogenase, which is usually used to establish the insomnia rat model. In our results, PCPA can induce a dose-dependent increase in moving duration, which could be effectively relieved by applying melatonin. Using this novel insomnia model, we successfully certificated the anti-insomnia effects of the PAMs of GABAARs screened from HWD. This novel model may help to establish a drug screen system to identify novel sleeping aids.

## Data Availability

The original contributions presented in the study are included in the article/Supplementary Material, further inquiries can be directed to the corresponding authors.

## Abbreviations

GABAAR, γ-aminobutyric acid type A receptor; HWD, Huanglian Wendan Decoction; TCM, traditional Chinese medicine; LC-MS, liquid chromatography-mass spectrometry; TCMSP, Traditional Chinese Medicine Systems Pharmacology; DRC, dose-response curve; PAM, positive allosteric modulator; BDZ, Benzodiazepine; PCPA, 4-Chloro-DL-phenylalanine; HL, Huanglian, Coptis chinensis Franch; BX, Banxia, Pinellia ternata (Thunb.); ZS, Zhishi, Citrus × aurantium L; CP, Chenpi, Citrus reticulata Blanco; FL, Fuling, Smilax glabra Roxb; ZR, Zhuru, Bambusa tuldoides Munro; GC, Gancao, Glycyrrhiza glabra L; PDB, Protein Data Bank.
