# Supplementary material for: Activation of GABA type A receptor is involved in the anti-insomnia effect of Huanglian Wendan Decoction
Source: Front Pharmacol. 2024 May 23;15:1389768. doi: 10.3389/fphar.2024.1389768 (PMC11153716; doi:10.3389/fphar.2024.1389768)
Supplement: Supplementary file 1 [file DataSheet1.docx]

**Supplementary materials**

**Table. S1 Main metabolites identification in HWD by LC-MS (QToF) in negative ion mode**

**Table. S2 Main metabolites identification in HWD by LC-MS (QToF) in positive ion mode**

**Figure. S1 The subtype selectivity of HWD.**

The effects of HWD on (A) α2β3γ2L, (B) α3β3γ2L, and (C) α6β3γ2L. (D) Statistic graph of the effects of HWD on different GABAARs.

**Figure. S2 The effects of 7 herbs on α1β3γ2L GABAARs.**

(A) Representative traces and (B) statistic graph of the effects of 7 herbs on α1β3γ2L GABAARs

**Figure. S3 The subtype selectivity of β-Caryophyllene, (+)-Cuparene, and Ethyl glucoside.**

Representative traces and statistic graph of (A) β-Caryophyllene, (B) (+)-Cuparene, and (C) Ethyl glucoside on different GABAARs. The α subunit was expressed with β3 and γ2L subunits.

**Figure. S4 The effects of Menthol and Stigmasterol on α1β3γ2L GABAARs at a concentration of 300 μM.**

**Table. S1 LC-MS (QToF) in negative ion mode**

| **Peak** | **RT** | **Mass** | **Name** | **DB Formula** | **DB Diff (ppm)** |
| --- | --- | --- | --- | --- | --- |
| 1 | 1.556 | 332.0846 | 1-Hydroxy-2,3,4,7- tetramethoxyxanthone | C17 H16 O7 | 14.95 |
| 2 | 1.591 | 120.0416 | Methyl allyl tetrasulfide | C4 H8 O4 | 5.21 |
| 3 | 1.612 | 90.0306 | Lactic acid | C3 H6 O3 | 11.7 |
| 4 | 1.648 | 192.064 | D-(-)-Quinic acid D-(-)- | C7H12O6 | -3.07 |
| 5 | 1.852 | 192.0264 | Isocitric acid d | C6 H8 O7 | 3.1 |
| 6 | 1.861 | 116.0093 | Fumaric acid | C4 H4 O4 | 14.15 |
| 7 | 1.863 | 134.020  1 | Malic acid | C4 H6 O5 | 10.52 |
| 8 | 2.155 | 291.0943 | Sarmentosin epoxide | C11 H17 N O8 | 4 |
| 9 | 2.41 | 288.0831 | Dianthoside | C12 H16 O8 | 5.05 |
| 10 | 2.887 | 118.0267 | Succinic acid | C4 H6 O4 | -0.5 |
| 11 | 3.13 | 162.0521 | Danshensu | C6 H10 O5 | 4.69 |
| 12 | 6.113 | 198.0522 | Salvianic acid A | C9H9NaO 5 | 110993.9 |
| 13 | 6.955 | 154.0261 | Protocatechuic acid | C7 H6 O4 | 3.26 |
| 14 | 8.556 | 268.0573 | Homocystine | C8 H16 N2 O4 S2 | -8.12 |
| 15 | 9.937 | 368.1092 | Methyl chlorogenate | C17 H20 O9 | 4.03 |
| 16 | 10.356 | 486.137 | Cassigerol E | C28 H22 O8 | -11.34 |
| 17 | 11.072 | 594.1577 | Oroxin B | C27 H30 O15 | 1.33 |
| 18 | 11.202 | 341.1612 | Cularine | C20 H23 N O4 | 4.47 |
| 19 | 13.284 | 428.1676 | Bruceine F | C20 H28 O10 | 1.56 |
| 20 | 13.915 | 550.1677 | Angustiamarin | C26 H30 O13 | 1.65 |
| 21 | 13.979 | 604.251 | 9(betaH)-9-Dihydro- 19-acetoxy-10- deacetylbaccatin III | C31 H40 O12 | 1.7 |
| 22 | 15.2 | 580.18 | Liquiritigenin-7,4'- diglucoside | C27 H32 O14 | -1.44 |
| 23 | 15.746 | 558.1329 | (+)-Gallocatechin- hexacetate | C27 H26 O13 | 8.01 |
| 24 | 16.127 | 610.1898 | Mesuein | C28 H34 O15 | 0 |
| 25 | 16.364 | 339.1458 | Papaverine | C20 H21 N O4 | 3.72 |
| 26 | 20.09 | 594.1936 | Isosakuranetin-7- rutinoside | C28 H34 O14 | 2.11 |
| 27 | 22.903 | 272.0672 | Butein | C15 H12  O5 | 4.57 |
| 28 | 23.814 | 838.398 | Glyyunnanprosapogeni n D | C42 H62 O17 | 0.88 |
| 29 | 25.629 | 822.4037 | Glycyrrhizic acid | C42 H62 O16 | 0.12 |
| 30 | 26.645 | 470.1928 | Drummondin A | C26 H30 O8 | 2.65 |
| 31 | 34.791 | 256.2393 | 13-Methyl pentadecanoic acid | C16 H32 O2 | 3.72 |

**Table. S2 LC-MS (QToF) in positive ion mode**

| **Peak** | **RT** | **Mass** | **Name** | **DB Formula** | **DB Diff (ppm)** |
| --- | --- | --- | --- | --- | --- |
| 1 | 1.58 | 117.0787 | Betaine | C5 H11 N O2 | 2.63 |
| 2 | 1.645 | 129.0795 | 6xi-Methoxypiperidin-2-one | C6 H11 N O2 | -4.4 |
| 3 | 1.673 | 143.0954 | Dihydromyricetin | C15H12O8 | 1236642.3 |
| 4 | 1.999 | 149.084 | D-Cathinone | C9 H11 N O | 0.28 |
| 5 | 2.001 | 167.094  3 | Synephrine | C9 H13 N O2 | 1.86 |
| 6 | 3.618 | 179.0948 | N-Methylphenylalanine | C10 H13 N O2 | -0.76 |
| 7 | 9.579 | 313.1684 | 3-Epischelhammericine | C19 H23 N O3 | -1.81 |
| 8 | 11.235 | 341.1628 | Cularine | C20 H23 N O4 | -0.25 |
| 9 | 12.251 | 368.1115 | 3-O-Feruloylquinic acid | C17 H20 O9 | -2.15 |
| 10 | 13.3 | 335.0792 | Nandazurine | C19 H13 N O5 | 0.42 |
| 11 | 13.359 | 339.1475 | O-Methylbulbocapnine | C20 H21 N O4 | -1.39 |
| 12 | 13.562 | 351.1111 | Oxyberberine | C20 H17 N O5 | -1.09 |
| 13 | 13.697 | 355.1784 | Glaucine | C21 H25 N O4 | -0.18 |
| 14 | 14.763 | 323.1158 | Rutacridone epoxide | C19 H17 N O4 | -0.09 |
| 15 | 15.253 | 580.1795 | Narirutin | C27 H32 O14 | -0.49 |
| 16 | 15.799 | 272.0692 | Aromadendrin-5,7- dimethyl ether | C15 H12 O5 | -2.53 |
| 17 | 16.573 | 336.1241 | Epiberberine | C20H18NO4 | -1.4 |
| 18 | 16.696 | 302.0794 | Hesperetin | C16 H14 O6 | -1.03 |
| 19 | 16.704 | 337.128 | Jatrorrhizine | C20H19NO4 | 10.16 |
| 20 | 16.783 | 320.0927 | Coptisine | C19H15ClNO4 | 112393.14 |
| 21 | 17.53 | 367.1427 | Corynoline | C21 H21 N O5 | -1.9 |
| 22 | 18.091 | 333.1011 | Dihydrosanguinarine | C20 H15 N O4 | -2.99 |
| 23 | 18.476 | 351.15 | Palmatine | C21H21NO4 | -8.41 |
| 24 | 19.509 | 365.1631 | Tylophorinidine | C22 H23 N O4 | -1.14 |
| 25 | 19.885 | 439.143 |  | C28 H17N5 O |  |
| 26 | 20.007 | 349.1323 | Dihydrochelerythrine | C21 H19 N O4 | -2.5 |
| 27 | 20.033 | 469.153 |  | C28 H23N O6 |  |
| 28 | 20.132 | 469.1535 |  | C29 H19 N5 O2 |  |
| 29 | 20.373 | 455.1739 |  | C28 H25 N O5 |  |
| 30 | 20.706 | 485.184 |  | C29 H27N O6 |  |
| 31 | 22.056 | 724.2211 | Arillanin A | C33 H40 O18 | 0.49 |
| 32 | 23.439 | 242.0947 | Cearoin | C15 H14 O3 | -1.68 |
| 33 | 24.419 | 372.1208 | Isosinensetin | C20 H20 O7 | 0.18 |
| 34 | 26.182 | 260.1052 | Hemigossypol | C15 H16 O4 | -1.3 |
| 35 | 27.386 | 402.1316 | (3'R,4'R)-3'- Epoxyangeloyloxy-4'- acetoxy-3',4'- dihydroseselin | C21 H22 O8 | -0.36 |
| 36 | 28.069 | 432.1425 | 3,3',4',5,5',6,7-Heptamethoxyflavone | C22 H24 O9 | -1.14 |
| 37 | 28.428 | 372.1214 | 5,7,2',4',6'-Pentamethoxyflavone | C20 H20 O7 | -1.27 |

**Figure. S1**

**
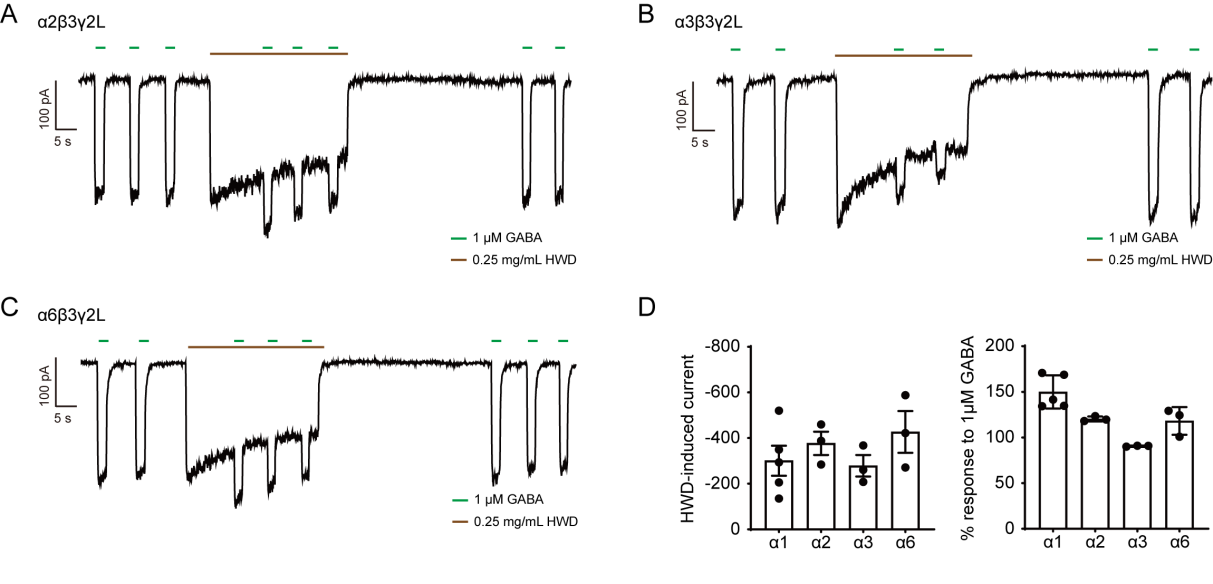
**

**Figure. S2**

**
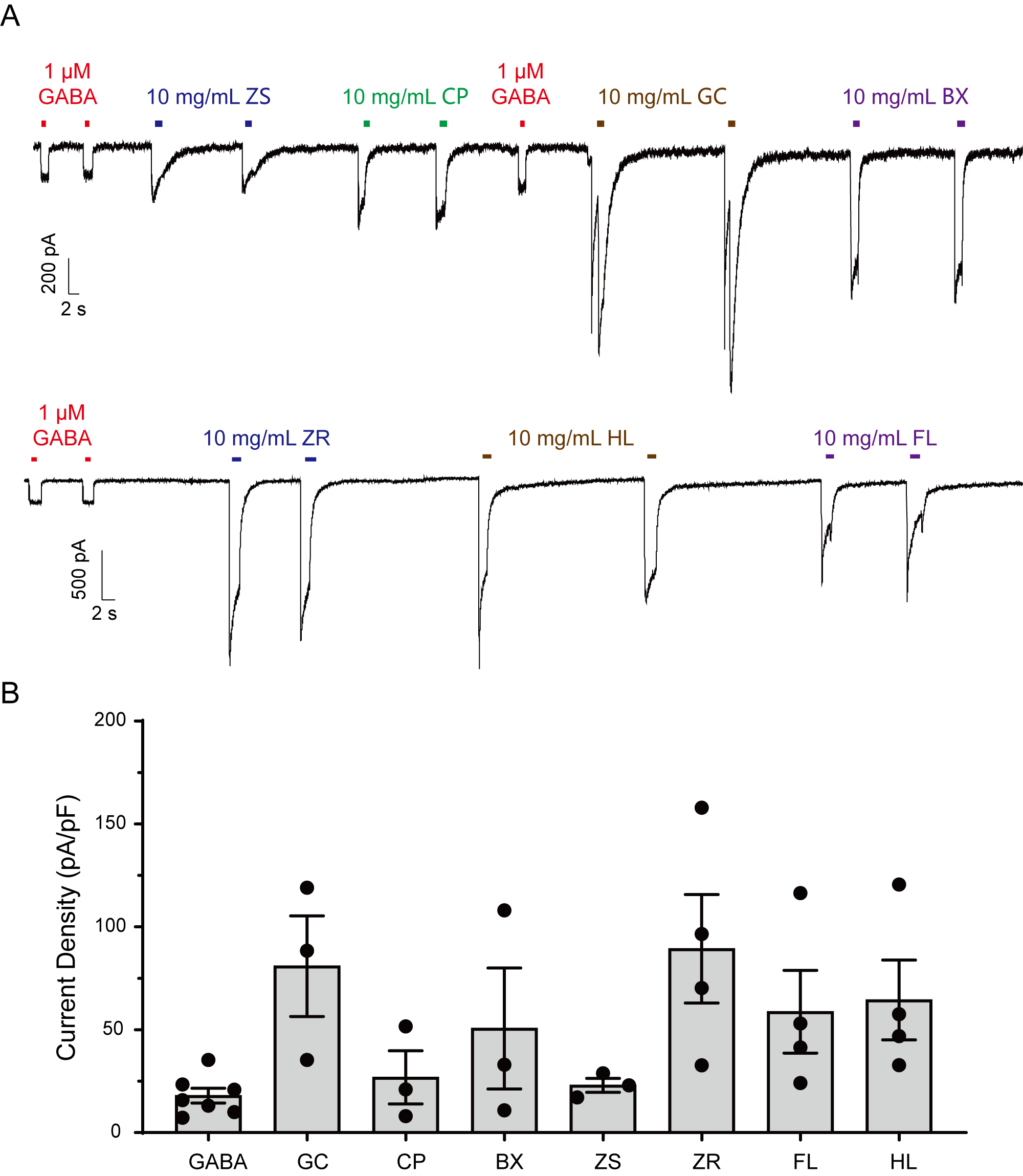
**

**Figure. S3**

**
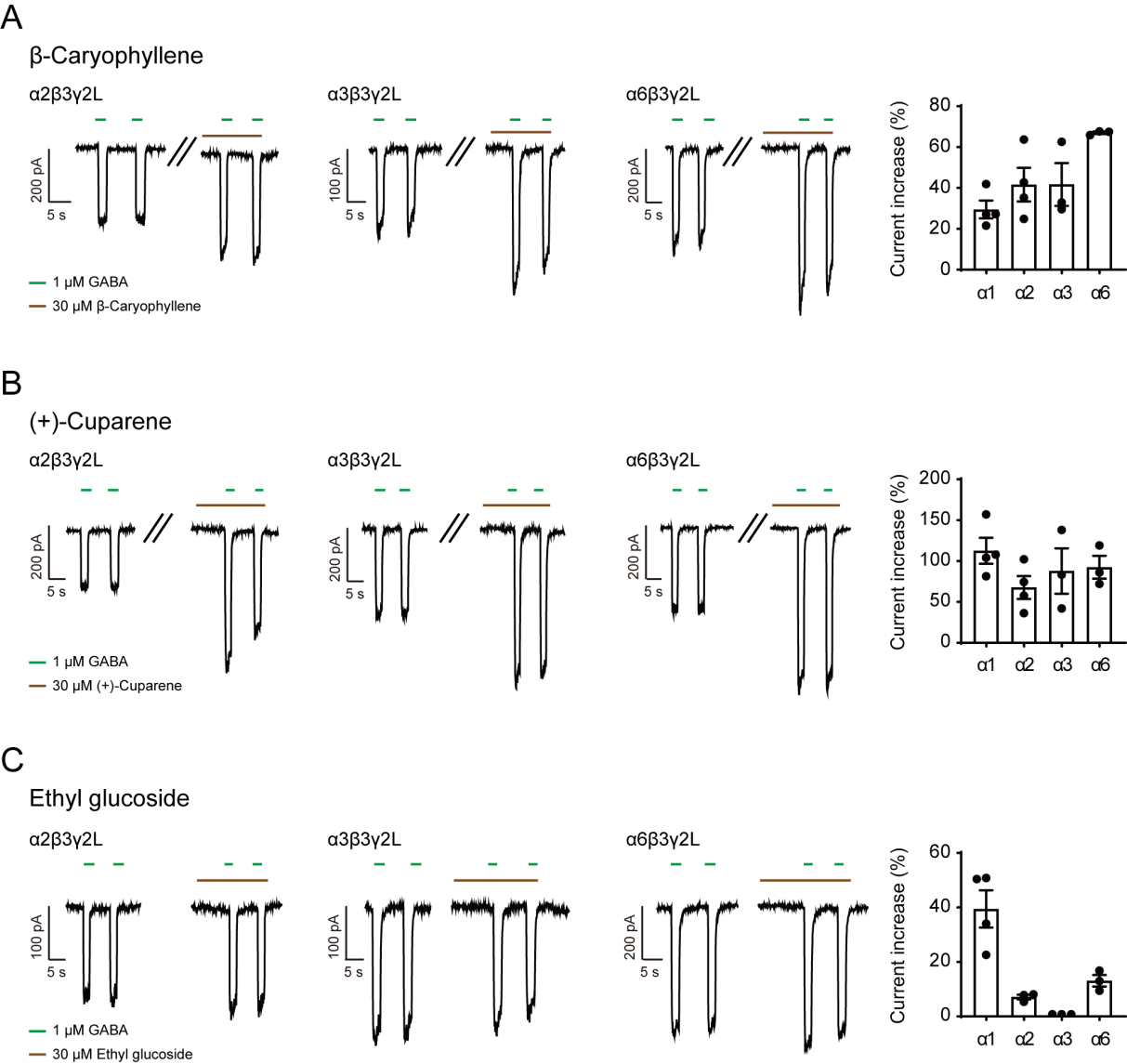
**

**Figure. S4**

**
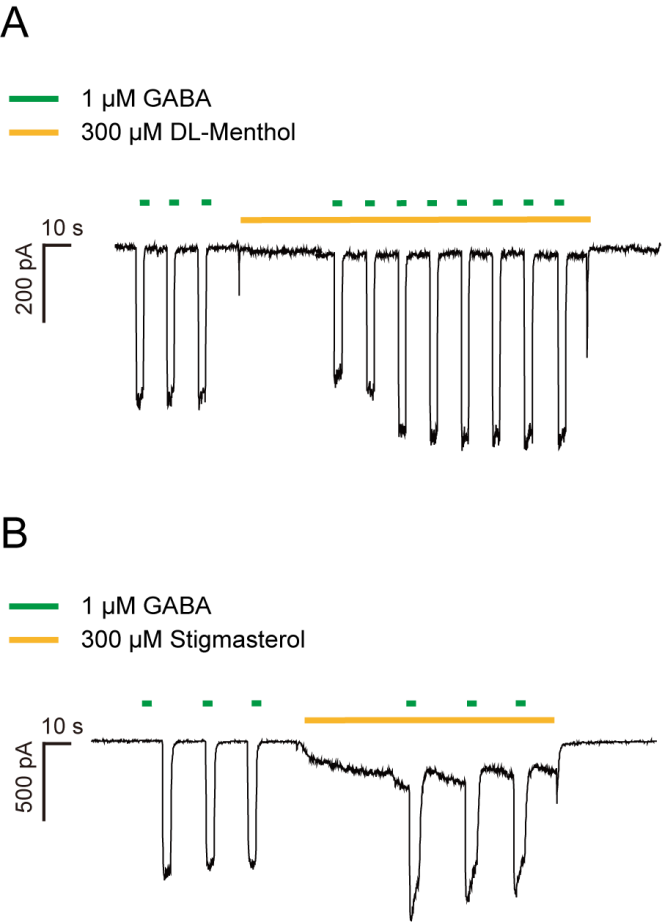
**

**Analysis of HPLC Fingerprint**

1. **Materials and Methods.**

(1) Optimization of the extraction process

An orthogonal experiment L_9_(3^4^) was designed and carried out, the factors under investigation including the amount of water (A), soaking time (B), boiling time (C), and number of boilings (D), each factor having 3 levels (Table. S3).

Table. S3 The factors and levels of the orthogonal design.

| Level | A  (Amount of water) | B  (Soaking time/min) | C  (Boiling time/h) | D  (Number of boilings) |
| --- | --- | --- | --- | --- |
| 1 | 10 | 0 | 0.5 | 1 |
| 2 | 12 | 30 | 1 | 2 |
| 3 | 14 | 60 | 1.5 | 3 |

(2) Preparation of extracts of Huanglian Wendan Decoction: Take 10 g of each herbs, including Huanglian (*Coptis chinensis Franch.*), Banxia (*Pinellia ternata (Thunb.)*), Zhishi (*Citrus × aurantium L.*), Chenpi (*Citrus reticulata Blanco*), Fuling (*Smilax glabra Roxb.*), Zhuru (*Bambusa tuldoides Munro*), and Gancao (*Glycyrrhiza glabra L*), into a round-bottom flask, add water according to the designed L_9_(3^4^) orthogonal array, soak, decoct, combine the decoction, filter, vacuum concentrate at 70°C, dilute with water to 50 ml, and obtain 9 batches of extract S1~S9. Additionally, prepare a reference extract solution by taking the corresponding dose of freeze-dried powder of Huanglian Wendan Decoction and preparing a solution.

(3) Preparation of reference solution: accurately weigh 0.5 mg of glycyrrhizin, 2 mg of hesperidin, 3 mg of neohesperidin, 5 mg of berberine, 3 mg of berberine hydrochloride, 5 mg of palmatine, and 5 mg of glycyrrhizic acid, place them in a 10 ml volumetric flask, add methanol to dissolve and dilute to the mark, shake well, filter through a 0.45 μm membrane filter, and collect the filtrate.

(4) Preparation of test solution: Take 2 ml of each of the above 9 batches of extracts (S1~S9) and 1 batch of reference extract (R), add methanol to 25 ml, seal, sonicate for 30 minutes, let stand, cool, shake well, filter through a 0.45 μm membrane filter, and collect the filtrate.

（5）Establishment and similarity evaluation of fingerprint chromatograms: analyze the reference and test solutions separately, record the chromatograms, and the chromatographic conditions refer to Table 2. Import the fingerprint chromatograms of the 9 batches of samples into *Similarity Evaluation System for Chromatographic Fingerprint of Traditional Chinese Medicine* sequentially. Use the chromatogram of the reference extract R as the reference. After multiple-point correction of the chromatographic peaks using the median method with a time window width of 0.10, match and generate a reference fingerprint chromatogram automatically (Figure 1).

Table. S4 Chromatographic condition.

| Chromatographic condition | Parameters | | |
| --- | --- | --- | --- |
| Chromatographic column | Zorbax SB C8，4.6*150mm，3.5um | | |
| Detector | DAD | | |
| Test wavelength | 242 | | |
| Column temperature | 45℃ | | |
| Mobile phase | A mobile phase：0.1%H3PO4 solution；B mobile phase：acetonitrile | | |
| Flow velocity | 1.0 ml/min | | |
| Elution program | Time（min） | Mobile phase（%） | Mobile phase |
|  | 0 | 80 | 20 |
|  | 4 | 65 | 35 |
|  | 21 | 58 | 42 |
|  | 26 | 58 | 42 |
|  | 29 | 45 | 55 |
|  | 33 | 45 | 55 |
|  | 38 | 10 | 90 |
|  | 55 | 10 | 90 |
|  | 55.1 | 80 | 20 |
|  | 60 | 80 | 20 |

| 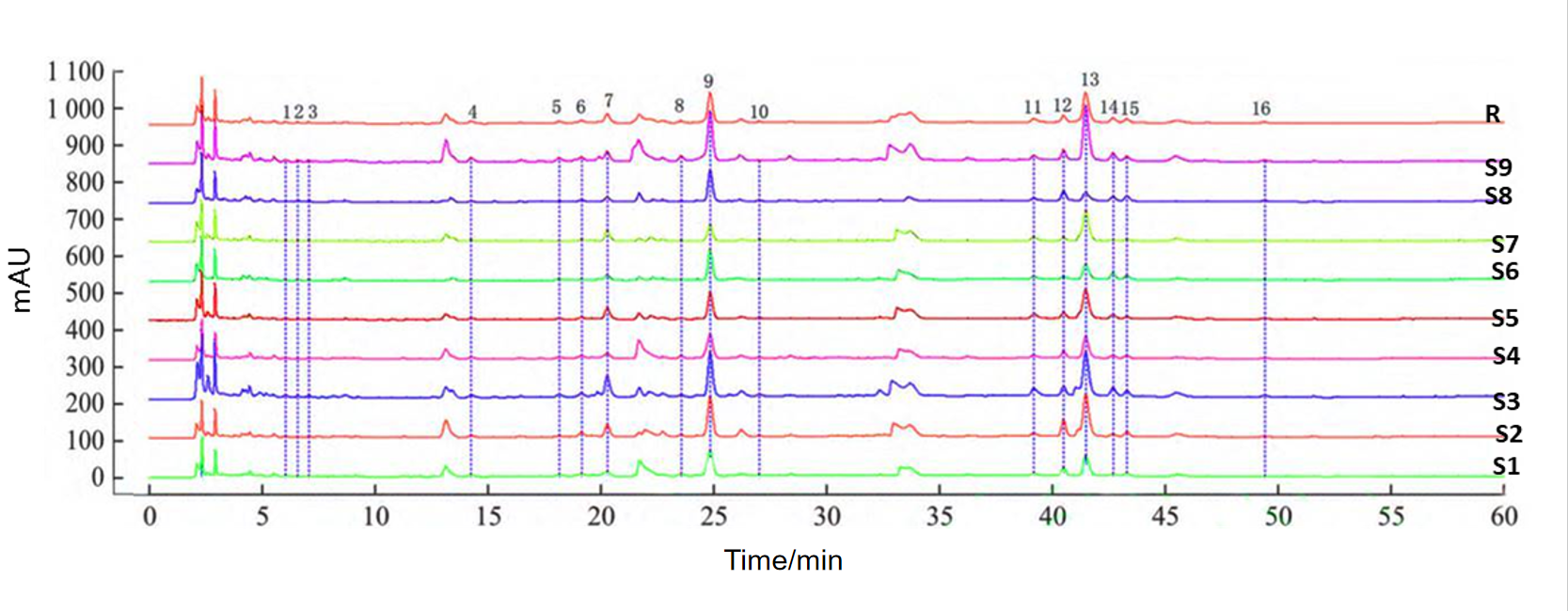 |
| --- |
| Fig. S5 HPLC fingerprints of 9 samples under different extraction conditions |

（6）Principal component data statistical analysis: The peak areas of the 16 common peaks in the HPLC fingerprint chromatograms of the 9 batches of Huanglian Wendan Decoction were used as features to obtain the original data matrix. SPSS 20.0 analysis software was used to perform cluster analysis and factor analysis.

1. **Results**

Sixteen characteristic common peaks (1–16) were selected in the fingerprints. Seven compounds were identified by comparing the retention time with standards, including liquiritin (5), hesperidin (7), neohesperidin (9), epiberberine (11), coptisine (12), berberine (13), glycyrrhizic acid (16) (Fig. 2).

Similarity values were calculated by comparing HPLC fingerprints of 9 samples with the reference fingerprints, the similarity values were 0.996, 0.994, 0.995, 0.996, 0.995, 0.997, and 0.997, respectively, suggesting good similarities.

| 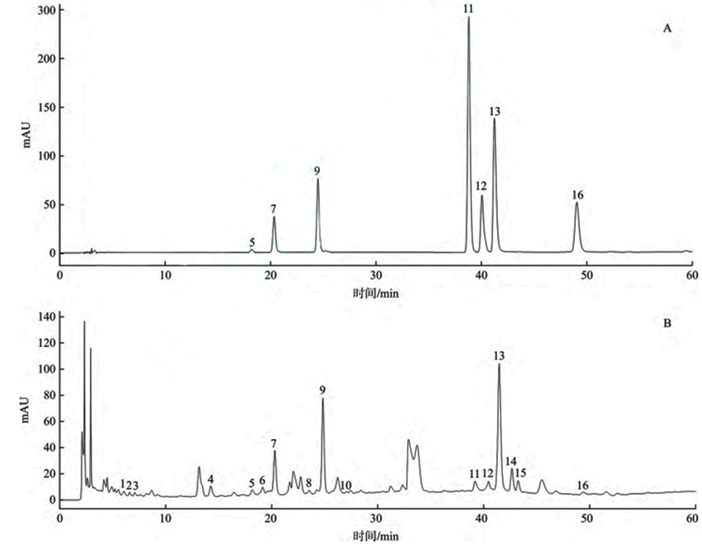 |
| --- |
| Fig. S6 HPLC fingerprints of Huanglian Wendan Decoction |

Referring to the Pharmacopoeia of People’s Republic of China (Part I, 2020 edition), berberine, coptisine, and palmatine are indicative components of the monarch drug Huanglian; hesperidin is an indicative component of the minister drug Chenpi; glycyrrhizin and glycyrrhizic acid are indicative components of the assistant drug Gancao. Therefore, the contents of these 6 components were used as indicators for weighted scoring. The scores of each indicator were multiplied by the weighting coefficient and then added to obtain the comprehensive score of the extraction process. The weighting coefficients for berberine, coptisine, palmatine, hesperidin, glycyrrhizin, and glycyrrhizic acid are 0.25, 0.25, 0.25, 0.15, 0.05, and 0.05, respectively. The final comprehensive score is shown in Tables 3 and 4.

From the orthogonal experiment analysis shown in Table 3, it can be visually analyzed that the degree of influence of factors is in the order of D > C > A > B, and the different levels of each factor are A1 > A3 > A2, B2 > B3 > B1, C3 > C1 > C2, D2 > D1 > D3. Taking the factor with the smallest degree of influence as the error, further analysis based on the variance analysis results in Table 4 shows that the amount of water (A), soaking time (B), boiling time (C), and number of boilings (D) have no significant effect on the extraction process (P >0.05). However, soaking time B and number of boilings D both show K2-K1 >> K3-K2, indicating that soaking time and number of boilings should both be at level 2. Considering aspects such as production cycle and cost, the final choice for the extraction process is A1B2C1D2, that is, 10 times the amount of water, soaking for 30 minutes, boiling twice, 1 h for each time.

Table. S5 Experimental design and results

| NO. | A  (Amount of water) | B  (Soaking time /min) | C  (Boiling time /h) | D  (Number of boilings) | Synthesis score |
| --- | --- | --- | --- | --- | --- |
| 1 | 1 | 1 | 1 | 1 | 0.08646 |
| 2 | 1 | 2 | 2 | 2 | 1.2262 |
| 3 | 1 | 3 | 3 | 3 | 0.9898 |
| 4 | 2 | 1 | 2 | 3 | -2.8061 |
| 5 | 2 | 2 | 3 | 1 | 0.4658 |
| 6 | 2 | 3 | 1 | 2 | 0.7407 |
| 7 | 3 | 1 | 3 | 2 | 0.9663 |
| 8 | 3 | 2 | 1 | 3 | 0.1162 |
| 9 | 3 | 3 | 2 | 1 | -0.0183 |
| K1 | 0.466 | -0.133 | 0.309 | 0.196 |  |
| K2 | -0.122 | 0.399 | -0.121 | 0.555 |  |
| K3 | 0.405 | 0.402 | 0.498 | -0.161 |  |
| R | 0.618 | 0.429 | 0.595 | 0.696 |  |

Table. S6 Analysis of variance

| Factor | Sum of squared deviations | Degree of freedom | *F-*ratios | Critical value |
| --- | --- | --- | --- | --- |
| A | 0.574 | 2 | 1.066 | 19.000 |
| B | 0.566 | 2 | 1.050 | 19.000 |
| C | 0.625 | 2 | 1.159 | 19.000 |
| D | 0.878 | 2 | 1.629 | 19.000 |
| Error | 0.567 | 2 |  | 19.000 |

NOTE：*F*_0.05_(2,2)=19.00，*F*_0.01_(2,2)=99.00[Note:*F*_0.05_(2,2)=19.00，*F*_0.01_(2,2)=99.00]
